# The association between emotional eating and depressive symptoms: a population-based twin study in Sri Lanka

**DOI:** 10.1017/gheg.2019.3

**Published:** 2019-05-08

**Authors:** Moritz P. Herle, Carol Kan, Kaushalya Jayaweera, Anushka Adikari, Sisira Siribaddana, Helena M.S. Zavos, Milana Smolkina, Athula Sumathipala, Clare Llewellyn, Khalida Ismail, Matthew Hotopf, Janet Treasure, Frühling Rijsdijk

**Affiliations:** 1 UCL Great Ormond Street Institute of Child Health, University College London, London, UK; 2 Section of Eating Disorders, Department of Psychological Medicine, Institute of Psychiatry, Psychology and Neuroscience, King's College London, London, UK; 3 Institute for Research and Development, Colombo, Sri Lanka; 4 Department of Medicine, University of Rajarata, Anuradhapura, Sri Lanka; 5 Social Genetic and Developmental Psychiatry Research Centre, Institute of Psychiatry, Psychology & Neuroscience, King's College London, London, UK; 6 National Addiction Centre, Institute of Psychiatry, Psychology, and Neuroscience, King's College London, London, UK; 7 Faculty of Medicine & Health Sciences, Institute for Primary Care & Health Sciences, , Keele University, Keele, UK; 8 Department of Behavioural Science and Health, University College London, UK; 9 Department of Psychological Medicine, Institute of Psychiatry Psychology and Neuroscience, King's College London, London, UK; 10 South London and Maudsley NHS Foundation Trust, London, UK

**Keywords:** Depression, emotional eating, global health, non-western population, twin research

## Abstract

This study investigated the genetic and environmental contributions to emotional overeating (EOE) and depressive symptoms, and their covariation, in a Sri-Lankan population, using genetic model-fitting analysis. In total, 3957 twins and singletons in the Colombo Twin and Singleton Study-Phase 2 rated their EOE behaviour and depressive symptoms, which were significantly associated (men: *r* = 0.11, 95% confidence interval (CI) 0.06–0.16, women: *r* = 0.12, 95% CI 0.07–0.16). Non-shared environmental factors explained the majority of variance in men (EOE *e*^2^ = 87%, 95% CI 78–95%; depressive symptoms *e*^2^ = 72%, 95% CI 61–83%) and women (EOE *e*^2^ = 76%, 95% CI 68–83%; depressive symptoms *e*^2^ = 64%, 95% CI 55–74%). Genetic factors were more important for EOE in women (*h*^2^ = 21%, 95% CI 4–32%) than men (*h*^2^ = 9%, 95% CI 0–20%). Shared-environmental factors were more important for depressive symptoms in men (*c*^2^ = 25%, 95% CI 10–36%) than women (*c*^2^ = 9%, 95% CI 0–35%). Non-shared environmental factors explained the overlap between depressive symptoms and EOE in women but not in men. Results differed from high-income populations, highlighting the need for behavioural genetic research in global populations.

## Introduction

Low mood and anhedonia (loss of pleasure of everyday activities) are core symptoms of depression. Other symptoms of depression include poor concentration, feeling of worthlessness, changes in sleep and appetite. Epidemiological studies have reported strong cross-sectional associations between depression and obesity; however, the direction of causation is poorly understood. Previous longitudinal meta-analyses have suggested a complex reciprocal relation between depression and obesity indicating causal links going from depression to weight gain and vice versa [1, 2]. Emotional overeating (EOE) is the tendency to respond to stress and negative emotions with food intake, and has previously been hypothesised as a behavioural mediator of the association between depression and weight gain. EOE has long been implicated as a key eating behaviour that predisposes to obesity [3], and it is well established that there are large individual differences in appetite changes in response to stress [4]. In addition, an association between EOE and depression was described in a cross-sectional study in a sample of 3714 Finnish adults. Both depressive symptoms and emotional eating were positively associated with unhealthy food choices, such as lower consumption of fruit and vegetables and increased consumption of sweets [5]. Higher rates of EOE amongst adults with comorbid for depression and obesity have also been reported [6].

The picture is further complicated by one longitudinal study that suggested that over a period of 5 years, EOE mediated the association between depression and weight gain for females only [7]; suggesting a sex difference in the aetiology between EOE, depression and weight gain. A stronger mediating effect in women of EOE for the depression-weight gain association has also been replicated in other European samples [8], highlighting the importance of incorporating sex differences in studies of the aetiology of EOE and depression. In addition to sex differences, analyses suggested that EOE played a mediating role in the association between depression and weight in unemployed participants *v*. employed participants, indicating that lower socioeconomic status and insecurity might be an important factor in these associations [8].

The twin method provides a powerful opportunity to assess the genetic and environmental aetiology of a complex trait within a particular population, as well as the common aetiology underlying two correlated phenotypes. This approach can deliver crucial insights into co-morbidity and furthers our understanding of complex disorders. So far majority of twin studies have focused on either depression [9] or EOE [10–14] and only one twin study has examined the common aetiological architecture underlying the two traits in a sample of healthy Korean adult twins suggesting differential genetic and environmental effects on EOE and depressive symptoms for men and women. Genetic effects for EOE were stronger in women than men, accounting for 40% and 30% of the EOE variation respectively [15]. EOE and depressive symptoms were correlated in this sample, and results suggested that both traits share some common genetic aetiology [15]. In addition, previous twin research has focused on understanding the shared aetiology between depression and eating disorders [16] as well as depressive symptoms and disordered eating behaviours commonly observed in patients with eating disorders, such as binge eating and self-induced vomiting [17]. However, these studies did not cover emotional eating which is observed in the healthy population [4]. Additionally, previous research has focused on high-income populations, with relatively low levels of social deprivation. Studying and comparing common disorders in low- to middle-income countries of non-Caucasian ancestry is crucial to test if findings derived from Western populations are truly generalisable. A study of depression in a large Sri Lankan sample of twins from the Colombo Twin and Singleton Follow-up Study (COTASS-2) [18] found a pervasive effect of the environment on individual differences in depression in men and stronger genetic effects in women, suggesting substantial differences in the aetiology of depressive symptoms in this population [19]. This study highlighted the importance of cross-cultural comparisons; because the environmental exposures associated with EOE and depression are likely to be specific to the cultural, economic and cultural context of the specific samples studied.

We therefore undertook the first twin study investigating the association between EOE and depressive symptoms using COTASS-2, a low-income population. The study had three objectives: (i) establish the association between EOE and depression in a non-European sample from a low-income country; (ii) estimate the genetic and environmental influences on EOE and depressive symptoms in this understudied population and (iii) investigate the extent of common genetic and environmental aetiology underlying both depressive symptoms and EOE.

## Methods

The study received ethical approval from Psychiatry, Nursing & Midwifery Research Ethics Subcommittee, King's College London, UK (reference number: PNM/10/11-124), and the Faculty of Medical Sciences University of Sri Jayewardenepura Ethical Review Committee (USJP ERC) (reference number: 596/11).

## Participants

COTASS was designed to investigate the relationship between metabolic diseases and mental health in the district of Colombo, Sri Lanka. The area is home to ~2.2 million people and is a mix of urban and rural areas. This study focuses on phase-2 of the study (COTASS-2), with a sample of 1647 twin pairs (for genetic model fitting) and 1035 singletons (included to estimate the phenotypic correlations) [20]. Demographic and phenotypic data were collected through extensive healthcare questionnaires.

## Measures

### Emotional overeating

Participants rated their EOE answering three questions adapted from the Three Factor Eating Questionnaire (TFEQ) [21] and the Dutch Eating Behaviour Questionnaire [22]. Participants used a Likert scale (ranging 0–4, never–always) to indicate if they generally experienced an upregulation in appetite when anxious (‘I eat more when I am anxious/nervous'), stress (‘I eat when things have gone wrong/are going against me) and boredom (‘I eat when I have nothing else to do’). Ratings were added up to create one overall score per participant (theoretical range: 0–12). Only participants who had answered all three questions were included.

### Depressive symptoms

Depressive symptoms were measured using the Beck Depression Inventory (BDI) which captures depressive symptoms and severity in the past 2 weeks [23]. Participants indicated on 21 items the extent to which the symptoms affected them, ranging from 0 (not at all) to 3 (severe) (theoretical range = 0–63). Raw scores of 0–13 indicate minimal depression, 14–19 indicate mild depression, 20–28 indicate moderate depression and 29–63 indicate severe depression [23]. The BDI was translated into Sinhalese by a panel of clinical professionals fluent in both Sinhalese and English. The BDI questionnaire was cross-culturally adapted in wording in order to best describe the questions in their meaning [24] and has been previously used in the Sri Lankan population [25].

### Zygosity and baseline measures

Zygosity of same-sex twin pairs was established by a self-report measure frequently used in twin research [26], which has undergone preliminary validation during the first wave data collection [27]. Socio-demographic information were extracted from the initial COTASS dataset.

### Analyses

The twin design is based on the comparison of monozygotic (MZ) and dizygotic (DZ) twins. MZ twins are natural clones, they share 100% of their DNA, whereas DZ twins share on average about half of their segregating genes. Importantly, as both types of twins are exposed to very similar environments, such as intrauterine exposures and parental upbringing, differences in similarity between MZ and DZ twin pairs are assumed to reflect genetic differences. Initially twin pair similarity is estimated separately for MZ and DZ twin using intraclass correlations, which indicate how similar two twins are on the observed phenotype (0: no similarity, 1: exactly the same). Further, twin designs allow a trait to be decomposed into three latent factors: (i) additive genetic influences (A); (ii) shared environmental influences (C), environmental factors that contribute to twin pair similarity above and beyond genetics (e.g. environmental factors affecting both twins in one family) and (iii) non-shared environmental factors (E), environmental factors that contribute to differences between twins within one pair (including random measurement error). Additionally the bivariate model allows for the covariance between two traits to be decomposed into A, C and E following the same principle. Applying a bivariate ACE twin model provides aetiological correlations (denoted *r*_A_, *r*_C_ and *r*_E_) which indicate the extent to which the A, C and E factors underlying individual differences for one trait also affect the other. Further, derived aetiological correlation can be used to decompose the phenotypic correlation between two traits. These bivariate estimates are calculated by dividing the aetiological correlations by the phenotypic correlation, deriving the proportion of the correlation that is due to latent factors A, C and E. Importantly, these bivariate estimates can only be calculated if *r*_A_, *r*_C_ and *r*_E_ are all positive or all negative [28].

Additionally twin modelling allows one to test for sex differences in the aetiology of traits. Sex differences are indicated by differences in twin correlations between same-sex and opposite-sex twin pairs [28]. The inclusion of same-sex and opposite-sex DZ twins enables testing for (i) qualitative sex differences – different genetic and environmental factors underlie variation and covariation for males and females; and (ii) quantitative sex differences – the same genetic and environmental factors underline variation and covariation for males and females but *differ in magnitude*. To identify the best fitting model, differences in minus twice the log-likelihood of (−2LL), similar to a χ^2^ test, were assessed, as well the Akaike's information criterion (AIC). Lower AIC values indicate a better model fit. Comparing the AIC of two models, a difference of 4–7 indicates support of one model over the other. An AIC difference of 10 indicates substantial support for the more parsimonious model [29]. Genetic model fitting was conducted using the statistical package OpenMX in R [30].

Scores on the BDI and EOE scale were used as continuous variables and regressed by age and sex, to account for the fact that age and sex, in same-sex twin pairs only, are completely shared within twin pairs. In addition, scores were log transformed to remove negative skew.

## Results

### Descriptive statistics

The characteristics of this sample are included in detail elsewhere [31]. The sample included in these analyses consisted of 3957 individuals (1680 males, 42.35%, and 2277, 57.65% females) with a mean age of 42.8 (s.d. 14.6) (see Table 1). Overall EOE behaviour was common, with one-fifth of the sample reporting to engage in emotional eating at least once, and scores ranged between 0 and 9 (theoretical maximum 12). The overall sample mean for the BDI was 4.86 (s.d. = 6.19) and scores ranged between 0 and 53 (theoretical maximum 63); 5% met the criteria for mild depression, 3% for moderate depression and 1% for severe depression. For full description of the distribution per EOE question and categories by severity of depression, see online Supplementary Table S1. Phenotypic correlations between EOE and depression were significant (males: 0.11, 95% confidence interval (CI) 0.06–0.16; females: 0.12, 95% CI 0.07–0.16).

**Table 1.** Descriptive statistics of samples included in analyses split by males and females

	Zygosity	Males	Females	Total number of individuals
Number of paired twins	MZ	478	668	1263
Number of single twins		55	62	
Number of paired twins	DZ	302	410	850
Number of single twins		63	75	
Number of paired twins	DZOS	343	343	809
Number of single twins		47	76	
	Singletons	392	643	1035
	Total	1680	2277	3957
Age Mean (s.d.)	MZ	37.53 (12.49)	39.32 (12.83)	
	DZ	39.41 (13.02)	43.09 (14.07)	
	DZOS	40.28 (13.19)		
	Singletons	52.17 (15.67)	50.65 (14.47)	
Depressive symptoms score^a^ Mean (s.d.) Range: 0–53	MZ	3.66 (5.18)	4.63 (5.71)	
	DZ	3.87 (5.49)	5.05 (6.14)	
	DZOS	4.63 (6.05)		
	Singletons	4.89 (6.29)	6.71 (7.31)	
Emotional eating score Mean (s.d.) Range: 0–12	MZ	0.64 (1.35)	0.59 (1.36)	
	DZ	0.48 (1.09)	0.47 (1.17)	
	DZOS	0.49 (1.18)		
	Singletons	0.28 (0.79)	0.23 (0.72)	
Emotional over-eating Intraclass correlations (95% CIs)	MZ	0.12 (0.02, 0.22)	0.26 (0.18, 0.33)	
	DZ	0.06 (−0.01 to 0.22)	0.05 (−0.01 to 0.17)	
	DZOS	0.14 (0.04–0.24)		
Depressive symptoms intraclass correlations (95% CIs)	MZ	0.29 (0.15–0.41)	0.36 (0.26–0.46)	
	DZ	0.24 (0.08–0.38)	0.22 (0.08–0.35)	
	DZOS	0.11 (0.00–0.21)		
Cross twin cross trait correlations (95% CIs)	MZ	0.06 (−0.02 to 0.14)	0.01 (−0.06 to 0.07)	
	DZ	0.01 (−0.10 to 0.12)	0.00 (−0.10 to 0.09)	
	DZOS	−0.04 (−0.11 to 0.04)		

MZ, monozygotic; DZ, dizygotic; DZOS, dizygotic opposite sex.

a Depressive symptoms were measured using the BDI.

### Genetic model fitting

A full sex-limitation bivariate model was fitted to the data, testing for both quantitative and qualitative sex differences (first for A and then for C). There were no statistical differences in model fit between the model allowing for quantitative differences only and the model allowing for qualitative differences in A (Δχ^2^ = 2.36, ΔDf = 4, *p* > 0.1, AIC = 8771.436, ΔAIC = 6.102) or qualitative difference in C (Δχ^2^ = 1.89, ΔDf = 4, *p* > 0.1, AIC = 8771.436, ΔAIC = 6.102). Results suggest no overall qualitative sex differences underlying the aetiology of EOE and depression.

To test if there were significant differences in the magnitudes of A, C and E between males and females (quantitative sex differences), path estimates were equated across males and females (homogeneity model). This constraint resulted in a significant decline in fit in comparison with the model allowing quantitative sex differences (Δχ^2^ = 158.4569, ΔDf = 6, *p* < 0.01, AIC = 8917.892, ΔAIC = 146.456); suggesting that A, C and E differed in magnitude for males and females.

**Fig. 1.**
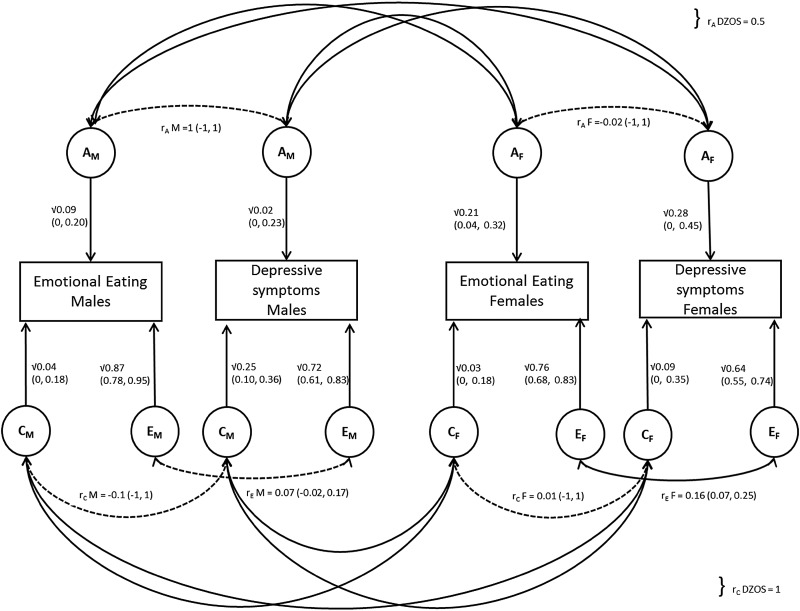
Path diagram illustrating bivariate ACE model allowing for quantitative sex differences; *r*_A_ and *r*_C_ for opposite sex (OS) DZ twins are fixed to 0.5 and 1, respectively. Latent factors are represented in circles for males (A_M_, C_M_, E_M_) and females (A_F_, C_F_, E_F_), with their path estimates as single headed arrows. All aetiological correlations were non-significant (indicated by a dotted line), apart from the one between the *E*_f_ factors in females (*r*_EF_).

Hence the best fitting model was the ACE bivariate sex-limitation model, allowing for quantitative sex differences only, indicating that the effects of A, C and E differ substantially between males and females. This model was also supported by the lowest AIC score (AIC = 8771.436). A path diagram of this model is shown in Fig. 1.

#### Decomposition of variance

Overall the majority of the variance was explained by non-shared environmental effects in males and females for both EOE (males: 87%, 95% CI 78–95%; females: 76%, 95% CI 68–83%) and depressive symptoms (males: 72%, 95% CI 61–83%; females: 64%, 95% CI 55–74%). Point estimates for genetic influence differed between males and females, with generally lower estimates in males (EOE = 9%, 95% CI 0–20%; depressive symptoms = 2%, 95% CI 0–23%) than in females (EOE = 21% 95% CI 4–32%; depressive symptoms = 28%, 95% CI 0–45%). The reverse was true for point estimates for shared-environmental factors which were higher in males, especially for depressive symptoms (25%, 95% CI 10–36%) in comparison with females (9%, 95% CI 0–35%).

CIs were wide for all estimates, due to the small sample sizes in the different sex-by-zygosity subgroups. The aetiological correlations in males were all individually non-significant, suggesting that the observed correlation between EOE and depressive symptoms in men is an accumulation of small, undetectable effects of *r*_AM_, *r*_CM_ and *r*_EM_.

#### Decomposition of covariance

The majority of the phenotypic correlation between depressive symptoms and EOE in males (*r* = 0.11) was mainly explained by non-shared environmental effects (55% = 0.06/0.11), whereas genetic effects (27% = 0.03/0.11) and shared environmental factors only explained 18% (0.02/0.11) attributed similarly. For females, only *r*_EF_ was significant (0.16, 95% CI 0.07–0.25) and non-shared environmental factors explained almost all of the phenotypic correlation between depressive symptoms and EOE (93% = 0.11/0.12). Both genetic factors (5% = 0.006/0.12) and shared-environmental factors (2% = −0.002/0.12) contributing less than 5%. The estimate for the contribution of shared-environment is negative, but very close to zero, so this partitioning of the phenotypic correlation in females is less reliable. See online Supplementary Table S2 for a full list for all results.

## Discussion

To our knowledge, this is the first twin study exploring the association between EOE and depressive symptoms in a low-income country and second one in any population. Furthermore, this study adds to the limited literature examining the aetiology of EOE, being the first twin study investigating this behaviour in a non-Western population.

Overall, one-fifth of the sample reporting that they engage in EOE to some extent, which is less common than in Western twins [10]. Regarding depressive symptoms, a previous study in this sample has reported a low prevalence of depression in Sri Lankan twins [32]. Similarly, here participants’ ratings of depressive symptoms were lower than in comparison with western populations [33, 34]. At a phenotypic level, EOE and depressive symptoms were positively correlated in males and females (*r*_M_ = 0.11, *r*_F_ = 0.12), although the sizes of the associations were small, and somewhat smaller than previous studies from Western populations (*r* = 0.31–0.44) [5–8].

Results from genetic modelling demonstrated small genetic effects on depressive symptoms in males, as reported for this sample previously [19]. Heritability of depressive symptoms was higher in women. For males, shared environmental factors were significant for depression. In contrast, for females, shared environmental factors played no role in individual differences in depressive symptoms. In comparison, estimates from twin studies in high-income Western countries, mostly report similar higher genetic effects for males and females, as well as no significant effect of the shared environment [35].

Regarding EOE, results confirmed previous studies from high-income countries, highlighting the importance of non-shared environmental factors in individual differences in EOE. Similar to depression, genetic factors underlying EOE were significant for females in comparison with males, which corroborates previous studies [12, 15]. In line with previous findings of adult twins, shared environmental factors were not significant for EOE. Overall, the majority of individual differences in adult EOE was found to be explained by non-shared environmental factors, in this sample, as well as in Western populations [10, 12] and non-Western high-income countries [11, 15].

One of the biggest differences observed, is the significant effect of the shared environment on individual differences in depressive symptoms in males. Shared-environmental factors are defined as environmental influencers, which contribute to the similarity within twin pairs. Cultural specific environmental factors might therefore underlie depressive symptoms in Sri Lankan males. Pervasive aspects of the living conditions in the Colombo capital district, such urban overcrowding and unstable employment, are likely to affect both brothers of one twin pair to the same extent regardless of their genetic relatedness. For females, overall stronger genetic effects underlying EOE and depressive symptoms in comparison with males might be an indication of reduced variation in environmental exposures in this Sri Lankan sample. A uniform environment across a population increases estimated genetic effects, leading to a reduction of shared environmental effects. This idea of a ‘blanket effect’ has previously discussed in the context of clinical depression, suggesting that lower emancipation and opportunities for women in Sri Lankan society might result in a higher observed heritability [19].

Moving to the analyses of the association between EOE and depressive symptoms, estimated phenotypic correlations were small and equal for males and females. Aetiological correlations between EOE and depressive symptoms were mostly found to be non-significant. In females, non-shared environmental effects underlying EOE and depression explained the phenotypic correlation. This finding supports the pervasiveness of unique environmental stressors on the development of depressive symptoms and associated behaviours such as EOE. In males, none of the aetiological correlations reached significance, with all CIs crossing zero. Results are limited by the reduced sample size due to the stratification into subgroups by sex and zygosity, leading to unreliable results with non-significant parameters. Overall, findings suggest a potential important role for EOE in the development and maintenance of depression. However, these cross-sectional data, cannot establish if EOE is a cause or consequence of depressive symptoms, and future longitudinal studies in this population are needed. EOE has been found to be modifiable through mindfulness interventions in a Western sample; however, it is not known if these strategies would be successful in this population [36]. Additionally, the complex interplay between body mass index, EOE and depression has not been studied in this population, and future research should aim to disentangle the common aetiology of all three.

### Strengths and limitations

As all other twin research, this current study needs to conform to the assumptions underlying the twin method. ‘The Equal Environment Assumption’ (EEA) states that environmental exposures influencing the variation of a trait are unrelated to the zygosity of the twin pairs – i.e. that MZs and DZs share their environments to the same extent. A violation of the EEA could lead to an overestimation of the genetic contribution to variation. Previous studies have confirmed the validity of the EEA in twin studies in general [37], as well as specifically in twin research studying eating behaviours [38].

One limitation of the study is the use of self-report questionnaires. Even though psychometric questionnaires are common to facilitate large-scale quantitative genetic research, they are prone to introduce reporting biases. The use of questionnaires to quantify EOE behaviour has recently been criticised, as there is a lack of evidence for an association between self-rated EOE and objective energy intake, as well as the potential confounding factors related to EOE such as emotion regulation skills and negative affect [39, 40]. In addition, the adapted questionnaire used to assess EOE has not been formally validated in western or non-western populations. However, due to the absence of valid objective measures for EOE in developing countries and the infeasibility of large-scale laboratory testing to provide large sample sizes, psychometric questionnaires remain the most pragmatic choice. Further cross-cultural differences in their understanding of depressive symptoms as well as emotional eating might have been of influence here. A previous study comparing depressive symptoms reported between White-British and people with south Asian origin in the UK indicated that the latter was more likely to disclose somatic instead of psychological symptoms [41]. Overall, these limitations of the measures discussed above, potentially introduced error into the twin analyses, which might be reflected in the large effects of non-shared environmental factors.

Even though this study is one of the largest twin sample in a low- to middle-income country, the large CIs around the produced estimates indicate low power to detect precise effects. This is especially clear when splitting the sample by zygosity and sex to detect sex differences. Similar problems have been reported by previous twin studies trying to test for sex differences [12] and the sample analysed in this current study exceeds all previous twin studies of EOE in adults [10–12].

### Cross-cultural comparisons in behavioural genetic research

The largest meta-analysis of twin studies so far confirms an over representation of research into the aetiology of individual differences in developed countries [42]. The report highlights that even though twin studies are conducted worldwide, the majority of results are from data collected in high-income countries, and the continents South America, Africa and Asia are highly under-represented [42].

Together with studies previously conducted [19, 43], we confirm that estimates derived from twin modelling are different between low- to middle-income and high-income countries, highlighting that cross-cultural differences such as inequality and socio-economic deprivation can affect the aetiology of human behaviour and disease. Twin research from developing countries is scarce, but crucial, as findings from high-income countries do not always necessarily extrapolate across cultures.

## Supplementary material

For supplementary material accompanying this paper visit https://doi.org/10.1017/gheg.2019.3. click here to view supplementary material
