# Supplementary material for: The association between emotional eating and depressive symptoms: a population-based twin study in Sri Lanka
Source: Glob Health Epidemiol Genom. 2019 May 8;4:e4. doi: 10.1017/gheg.2019.3 (PMC6509968; doi:10.1017/gheg.2019.3)
Supplement: Supplementary file 1 [file S2054420019000034sup001.docx]

Supplementary Table 1: Distribution of answers for emotional eating questions

|  | **I eat more when I am worried/anxious** | | | | **I eat more when things have gone wrong** | | **I eat when I have nothing else to do** | | |
| --- | --- | --- | --- | --- | --- | --- | --- | --- | --- |
|  | | **N** | | **%** | **N** | **%** | **N** | | **%** |
| **never** | | 3,651 | | 92.81 | 3,764 | 95.68 | 3,349 | | 85.15 |
| **rarely** | | 145 | | 3.69 | 100 | 2.54 | 243 | | 6.18 |
| **sometimes** | | 87 | | 2.21 | 40 | 1.02 | 224 | | 5.7 |
| **often** | | 48 | | 1.22 | 24 | 0.61 | 102 | | 2.59 |
| **always** | | 3 | | 0.08 | 6 | 0.15 | 15 | | 0.38 |
| **Total** | | 3,934 | | 100 | 3,934 | 100 | 3,933 | | 100 |
|  |  | | |  |  |  |  | |  |
| **Sum score** | | | **N** | **%** | **Beck’s Depression Inventory – categories** | | | | |
| **0** | | | 3,140 | 79.84 |  | | | **N** | **%** |
| **1** | | | 283 | 7.2 | **Minimal depression (<14)** | | | 3,556 | 90.88 |
| **2** | | | 267 | 6.79 | **Mild depression (14-19)** | | | 209 | 5.34 |
| **3** | | | 128 | 3.25 | **Moderate depression (20-28)** | | | 112 | 2.86 |
| **4** | | | 50 | 1.27 | **Severe depression (29-63)** | | | 36 | 0.92 |
| **5** | | | 22 | 0.56 | **Total** | | | 3,913 |  |
| **6** | | | 24 | 0.61 |  |  |  | |  |
| **7** | | | 8 | 0.2 |  |  |  | |  |
| **8** | | | 2 | 0.05 |  |  |  | |  |
| **9** | | | 9 | 0.23 |  |  |  | |  |
| **Total** | | |  |  |  |  |  | |  |

Supplementary Table 2: Decomposition of variance and co-variance in emotional overeating and depressive symptoms separate by males and females

| **Males** | | | | | | |
| --- | --- | --- | --- | --- | --- | --- |
| **Decomposition of variance** | | | | | | |
|  | **A** | **95% CI** | **C** | **95% CI** | **E** | **95% CI** |
| **Emotional eating** | 9% | 0, 20 | 4% | 0, 18 | 87% | 78, % |
| **Depressive symptoms** | 2% | 0, 23 | 25% | 10, 36 | 72% | 61, 83 |
| **phenotypic correlation** | 0.11 | 0.06, 0.16 |  |  |  |  |
| **Decomposition of covariance** | | | | | | |
|  | **A** |  | **C** |  | **E** |  |
|  | 27% |  | 18% |  | 55% |  |
| **Females** | | | | | | |
| **Decomposition of variance** | | | | | | |
|  | **A** | **95% CI** | **C** | **95% CI** | **E** | **95% CI** |
| **Emotional eating** | 21% | 4, 32 | 3% | 0, 18 | 76% | 68, 83 |
| **Depressive symptoms** | 28% | 0, 45 | 9% | 0, 35 | 64% | 55, 74 |
| **phenotypic correlation** | 0.12 | 0.07, 0,16 |  |  |  |  |
| **Decomposition of covariance** | | | | | | |
|  | **A** |  | **C** |  | **E** |  |
|  | 5% |  | 2%* |  | 93% |  |

* Estimate for the contribution of shared environment was negative, but close to zero (2%= -0.002/0.12)
